# Healthy and unhealthy plant-based diet quality and whole-brain imaging-derived phenotypes: an imaging-wide association study

**DOI:** 10.1016/j.jnha.2026.101027

**Published:** 2026-11-03

**Authors:** Boding Wang, Kai Wang, Liyuan Han, Yiling Wang, Feiyu Chen, Chuyi Cai

**Affiliations:** a Department of Neurosurgery, Ningbo No.2 Hospital, Wenzhou Medical University, 41 Xibei Road, Haishui District, 315010 Ningbo, Zhejiang Province, China; b Department of Radiology, Ningbo No.2 Hospital, Wenzhou Medical University, 41 Xibei Road, Haishui District, 315010 Ningbo, Zhejiang Province, China

**Keywords:** Plant-based diet, Diet quality, Brain MRI, Imaging-derived phenotypes, UK Biobank

## Abstract

**Objectives:**

Plant-based diets are widely promoted for cardiometabolic and brain health, yet their associations with health may depend on the quality of the plant foods consumed. Whether healthy and unhealthy plant-based diet quality differ in their associations with brain structure across the whole brain remains unclear.

**Methods:**

In a cross-sectional analysis of 23,460 participants with 24 -h dietary recalls and brain MRI, we performed an imaging-wide association study of standardized healthy (hPDI) and unhealthy (uPDI) plant-based diet indices against 910 imaging-derived phenotypes (IDPs) spanning structural, diffusion, and functional MRI. Multivariable linear regression (adjusting for age, sex, ethnicity, household income, education, smoking, alcohol, physical activity, and total energy intake) yielded standardized beta coefficients; category-level and global false-discovery-rate (FDR) correction controlled multiple testing. The overall plant-based diet index (PDI), energy-adjusted Dietary Inflammatory Index (E-DII), DII, DASH, and AHEI served as comparison scores.

**Results:**

uPDI showed widespread associations, with 187 white-matter diffusion and 110 structural-volume IDPs reaching category-FDR < 0.05, versus 1 and 19, respectively, for hPDI. Higher uPDI was associated with greater normalized grey-matter volume (beta = 0.038, 95% CI 0.028 to 0.047) and higher fornix fractional anisotropy (beta = 0.029, 95% CI 0.018 to 0.040). hPDI and uPDI were weakly correlated (Pearson r = 0.230), and variance inflation factors were 1.16 and 1.13, respectively. In models including both indices, uPDI retained category-FDR-significant associations with 89 structural and 46 diffusion IDPs. The overall PDI and E-DII also showed widespread imaging associations; after mutual adjustment for overall PDI, uPDI retained associations with only 20 structural and 1 diffusion IDP.

**Conclusions:**

Unhealthy plant-based diet quality was associated with broad structural and white-matter imaging signatures that were substantially more numerous than those observed for healthy plant-based diet quality and remained evident after mutual adjustment for hPDI. Higher uPDI was associated with greater normalized grey-matter volume and higher fornix fractional anisotropy, a direction that was contrary to expectations based on prior literature. The findings from the overall PDI further indicate that much of this uPDI signal overlapped with overall plant-versus-animal dietary composition, leaving only a limited independent signal after mutual adjustment; these associations therefore cannot be attributed specifically to the quality of plant foods and warrant longitudinal confirmation.

## 1 Introduction

Plant-based diets have been increasingly promoted for both human and planetary health, and suboptimal diet is among the leading modifiable risk factors for noncommunicable disease and premature death worldwide [1]. Dietary guidance and influential commissions now recommend a shift toward predominantly plant-based, sustainable eating patterns [2], and meta-analyses of observational studies report that vegetarian and vegan diets are associated with lower risks of ischemic heart disease and total cancer [3]. However, plant-based eating is biologically heterogeneous, and the health implications of a diet defined only by its plant content are likely to depend on which plant foods predominate.

This recognition has motivated the use of plant-based diet indices that distinguish the nutritional quality of plant-derived foods rather than treating all plant foods as equivalent. The hPDI gives greater weight to foods such as whole grains, fruits, vegetables, legumes, and nuts, which are major sources of dietary fibre, polyphenols, micronutrients, and unsaturated fatty acids. In contrast, the uPDI places greater weight on refined grains, sugar-sweetened beverages, and sweets, which generally provide lower nutrient density and greater exposure to rapidly absorbed carbohydrates and added sugars. These differences are relevant to brain health because metabolic and vascular disturbances, systemic inflammation, and impaired vascular function may affect neuronal integrity and white-matter organization. Consistent with this framework, higher hPDI has been associated with more favorable cardiometabolic outcomes, whereas higher uPDI has been associated with adverse metabolic and vascular profiles [4,5]. Diet quality is therefore biologically plausible as a determinant of brain structure, with the nutritional composition of plant-based diets potentially influencing brain imaging phenotypes through interconnected metabolic, vascular, and inflammatory pathways [6,7].

Brain imaging-derived phenotypes (IDPs) provide quantitative intermediate measures of brain structure and microstructure that may capture diet-related differences before clinical disease emerges. Dietary factors have been linked to brain and cognitive health, with higher consumption of sugar-sweetened beverages associated with adverse brain and cognitive markers [8]. UK Biobank provides an opportunity to examine these relationships systematically because its standardized multimodal MRI resource includes a large number of structural, diffusion, and functional imaging-derived phenotypes [[9], [10], [11]]. Previous neuroimaging studies further suggest that overall dietary quality may be reflected in brain structure: greater adherence to Mediterranean-style dietary patterns has been associated with larger brain and grey-matter volumes [12,13], whereas unhealthy or Western dietary patterns have been linked to smaller hippocampal volume [14]. Although these findings do not establish a uniform effect across all imaging phenotypes, they provide a rationale for expecting healthier dietary composition to be associated with more favorable brain structural and white-matter characteristics. A brain-wide analysis can therefore assess whether these diet-brain relationships extend beyond individual regions selected in advance. However, previous diet-brain imaging studies have generally focused on a limited number of dietary scores or predefined brain regions, leaving the broader imaging phenome relatively unexplored. Large-scale, hypothesis-agnostic scans across hundreds of IDPs remain uncommon, despite the large sample sizes needed to obtain reproducible brain-wide associations [15]. In addition, dietary indices such as the Dietary Inflammatory Index [16], the Dietary Approaches to Stop Hypertension (DASH) diet [17], and the Alternative Healthy Eating Index (AHEI) [18] have been associated with systemic health outcomes but have rarely been evaluated systematically across the whole brain imaging phenome.

To date, no study has systematically compared healthy and unhealthy plant-based diet quality against the whole-brain imaging phenome. This gap is notable because a recent analysis linked a healthful plant-based diet to lower, and an unhealthful plant-based diet to higher, risk of dementia and depression, yet the neurostructural substrate of these associations is unknown [19]. We therefore performed an imaging-wide association study (IWAS) in UK Biobank, relating standardized hPDI and uPDI to 910 brain IDPs, with uPDI as the primary exposure and hPDI as the control exposure, and benchmarking both against the overall PDI, E-DII, DII, DASH, and AHEI scores. Our primary working hypothesis was that a higher hPDI score would be associated with more favorable brain structural and white-matter characteristics, whereas a higher uPDI score would generally be associated with less favorable characteristics. Our aims were to (1) quantify the imaging-wide associations of hPDI and uPDI across IDP categories under false-discovery-rate control; (2) identify the biologically interpretable structural and white-matter phenotypes most strongly associated with uPDI; and (3) assess the independence of hPDI and uPDI and benchmark their associations against additional dietary scores.

## 2 Materials and methods

### 2.1 Study design and data source

We performed a cross-sectional imaging-wide association analysis in participants who had both 24 -h dietary recall data and brain MRI imaging-derived phenotypes (IDPs) [20]. Dietary exposures were derived from the Oxford WebQ 24 -h dietary recall instrument [21], using plant-based diet indices computed in previous work. Brain outcomes were IDPs covering structural MRI volumes, diffusion MRI white-matter tract microstructure, functional MRI/T2star metrics, and additional image-quality or technical metrics, generated through the standardized FMRIB processing pipeline [9,10]. The study addressed differences in plant-based diet quality rather than a simple distinction between plant-based and non-plant-based diets. The primary exposure was the unhealthy plant-based diet index (uPDI) and the control exposure was the healthy plant-based diet index (hPDI); higher hPDI reflects greater intake of healthy plant foods and higher uPDI reflects greater intake of unhealthy plant foods. Reporting followed the Strengthening the Reporting of Observational Studies in Epidemiology (STROBE) guideline [22].

### 2.2 Study population

Participants were individuals with available diet scores, brain MRI IDP data, and key covariate information. The initial dietary covariate dataset contained 95,046 individuals and the imaging IDP dataset contained 83,984 individuals, with 28,634 individuals overlapping between diet and imaging. After excluding 523 individuals with major neurological disease (dementia, Parkinson's disease, multiple sclerosis, or stroke) based on available ICD-10 diagnoses, 28,111 individuals remained; after further excluding 4,651 individuals with missing hPDI, uPDI, or Model 2 covariates, the final hPDI/uPDI Model 2 analytic sample comprised 23,460 individuals (Fig. 1). Ethical approval was obtained from the North West Multi-centre Research Ethics Committee in the UK. All participants provided informed consent at enrolment. The current analyses were conducted under application 78619.

**Fig. 1:**
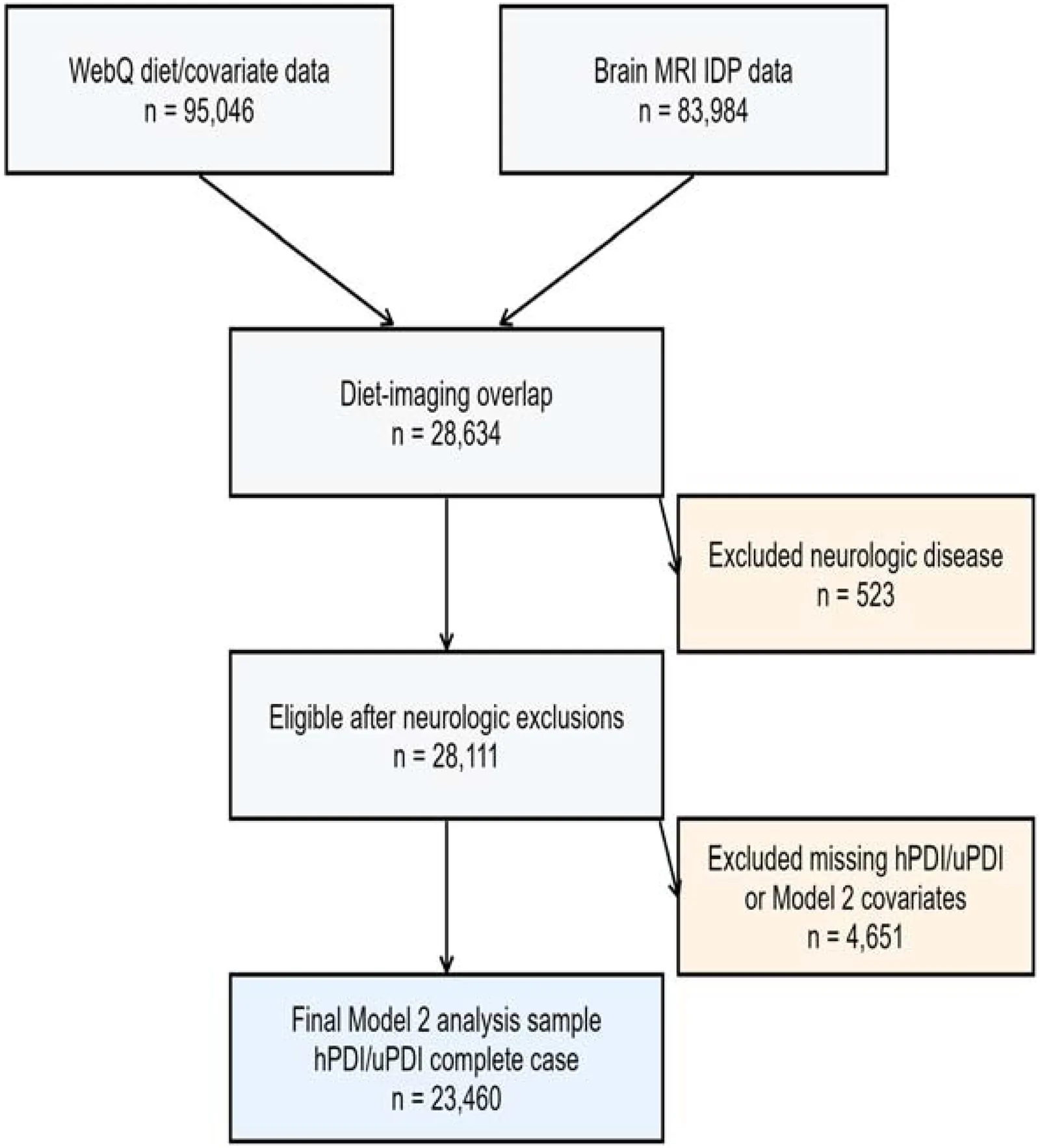
Flow of participant selection for the plant-based diet quality and whole-brain imaging-derived phenotype (IDP) analysis.

### 2.3 Dietary exposures

The UKB-adapted plant-based diet indices were constructed from 15 available food groups derived from Oxford WebQ dietary recalls. Healthy plant foods comprised whole grains, fruits, vegetables, nuts, and legumes; less-healthy plant foods comprised refined grains, potatoes, sugar-sweetened beverages, and sweets and desserts; and animal foods comprised red meat, processed meat, poultry, fish, eggs, and dairy. For each food group, intakes (servings/day) were classified into ordered categories from the lowest to the highest intake using empirical quintile cut points (the 20th, 40th, 60th, and 80th percentiles; R quantile type 7), which were derived in the full dietary score-construction cohort before restriction to the imaging analysis sample (n = 210,722). If empirical cut points were identical because of tied intake values, duplicate cut points were collapsed, so that participants with identical intake values were never split across categories; consequently, all recorded zero intakes were retained in the same lowest intake category and some components formed fewer than five distinct intake categories. Component-specific intake distributions and non-consumer placement across the actual scoring categories are provided in Supplementary Table S7. For hPDI, healthy plant foods were scored positively, whereas less healthy plant foods and animal foods were reverse-scored. For uPDI, less healthy plant foods were scored positively, whereas healthy plant foods and animal foods were reverse-scored. For PDI, higher intake of healthy and less healthy plant foods received higher component scores, whereas animal foods were reverse-scored. Points were then assigned according to the rank of the intake category without rescaling: for a positively scored food group, the kth category from the lowest intake received k points, whereas for a reverse-scored food group it received 6 − k points. Food groups with fewer than five categories therefore received only part of the 1–5 range; in uPDI, for example, whole grains received 5, 4, or 3 points, nuts 5 or 4 points, and sugar-sweetened beverages 1 or 2 points, and only fruits, vegetables, and refined grains spanned the full range (Supplementary Table S7). Participants without a recorded intake for a food group (for example, 9,217 participants for whole grains) were assigned a missing component score, and the missing component was omitted from the index sum. Component scores were then summed across the 15 food groups. Had every food group formed five categories, each index would have ranged from 15 to 75; because points were not rescaled, the attainable range of uPDI given the categories actually formed was 35–69, and the observed uPDI ranged from 14 to 49 in the analytic sample. Food groups with fewer categories spanned fewer points and could therefore contribute less to the variation in the summed score. Each index was then standardized to a z-score. Accordingly, effect estimates represent the difference in standardized IDP per one-standard-deviation increment in the diet score [4,5].

### 2.4 Brain imaging phenotypes

We analyzed 910 brain IDPs [11]. IDPs were grouped by imaging type into structural MRI volumes, diffusion MRI white-matter tract metrics, functional MRI/T2star metrics, and other imaging phenotypes. Technical or quality-control-related IDPs (such as image quality, head motion, scanner-table position, registration differences, and signal-to-noise ratio) were not interpreted as core biological outcomes, but their audit information was retained for results review and in the supplementary materials. Diffusion metrics such as fractional anisotropy index the orientational coherence of water diffusion in white matter and are sensitive to, but not specific for, the underlying tissue microstructure [23].

### 2.5 Covariates

The primary model (Model 2) adjusted for age, sex, ethnicity, household income, education, smoking status, alcohol drinking status, physical activity, and total energy intake. For structural MRI volume IDPs, the model additionally included T1 volumetric scaling as a head-size-related adjustment; when the scaling-related IDP itself was the outcome, it was not additionally included. Sensitivity models could further incorporate body mass index, diabetes, and hypertension, but the primary results reported here are based on Model 2.

### 2.6 Statistical analysis

All statistical analyses were performed in R (version 4.3.3). Baseline characteristics were summarized and compared across unhealthy plant-based diet index (uPDI) quartiles. Continuous variables are presented as median (interquartile range) and were compared with the Kruskal-Wallis H test; categorical variables are presented as n (%) and were compared with the Pearson chi-square test.

To characterize the dietary patterns underlying uPDI, mean food-group intakes and the proportion of non-consumers were summarized across uPDI quartiles; differences were evaluated using Kruskal–Wallis tests for intake distributions and chi-square tests for non-consumer proportions. To document score construction directly, we additionally summarized each food group across its own intake categories used for scoring, including the proportion of non-consumers within each category, the distribution of all non-consumers across categories, and the number of participants without a recorded intake. Pearson and Spearman coefficients quantified the correlation between hPDI and uPDI. Variance inflation factors were calculated from models containing both indices and the Model 2 covariates. To examine their independent associations, the imaging-wide models were repeated with hPDI and uPDI entered simultaneously. The overall PDI was analyzed as an additional comparison score using the same Model 2 specification and FDR procedures. Pearson and Spearman coefficients and variance inflation factors were also calculated for uPDI and overall PDI. To assess whether uPDI associations were independent of overall plant-versus-animal dietary composition, the imaging-wide models were repeated with uPDI and overall PDI entered simultaneously.

Imaging association analyses fitted a separate multivariable linear regression model for each brain IDP. All diet scores and imaging IDPs were standardized to z-scores, yielding standardized uPDI (uPDI_z) and standardized hPDI (hPDI_z). Therefore, regression coefficients represent the difference in standardized imaging phenotype per one-standard-deviation increment in dietary score and should be interpreted as relative associations rather than absolute changes in imaging measurements. Models were specified as: standardized IDP = standardized diet score + covariates, with uPDI_z as the primary exposure and hPDI_z as the control exposure.

Each regression model produced a regression coefficient (beta), standard error, 95% confidence interval, and raw P value. To control multiple-testing bias, we applied false-discovery-rate (FDR) correction, computing both a within-imaging-category FDR (category-FDR) and a whole-IDP FDR. Category-FDR < 0.05 was the primary significance threshold, and global-IDP FDR served as a stricter confirmatory criterion. To prevent technical imaging metrics from confounding the interpretation of true brain biological changes, the main text presents only structural and white-matter microstructure IDPs with clear biological interpretation; head motion, scanner-table position bias, image registration error, signal-to-noise ratio, and similar quality-control metrics were used for data quality control only and were not presented or analyzed as main-text results.

## 3 Results

### 3.1 Participants and baseline characteristics

The final hPDI/uPDI primary analytic sample comprised 23,460 individuals. The Q1, Q2, Q3, and Q4 uPDI quartiles contained 5,842, 5,834, 5,918, and 5,866 individuals, respectively (Table 1). uPDI levels increased across quartiles, with the median z-score rising from -0.72 in Q1 to 1.49 in Q4. The hPDI median also increased from -0.02 to 0.49, indicating that healthy and unhealthy plant-based diet scores varied partly in parallel in this sample rather than being mutually exclusive.

**Table 1:** Baseline characteristics by uPDI quartiles.

Characteristics	Q1 (lowest)	Q2	Q3	Q4 (highest)	P value
Age, years	56.00 (50.00, 61.00)	55.00 (49.00, 61.00)	55.00 (49.00, 61.00)	54.00 (48.00, 61.00)	<0.001
Physical activity, log MET-min/week	7.50 (6.80, 8.10)	7.48 (6.77, 8.07)	7.42 (6.70, 8.05)	7.37 (6.64, 8.03)	<0.001
Total energy intake, kJ/day	8320.94 (7035.39, 9828.54)	8482.46 (7233.22, 9913.77)	8598.65 (7399.30, 10010.71)	8783.35 (7558.87, 10156.97)	<0.001
Body mass index, kg/m2	25.63 (23.29, 28.52)	25.70 (23.33, 28.62)	25.73 (23.41, 28.47)	25.85 (23.49, 28.70)	0.053
hPDI, z-score	−0.02 (-0.53, 0.66)	0.32 (-0.36, 0.82)	0.49 (-0.19, 0.99)	0.49 (-0.02, 1.16)	<0.001
uPDI, z-score	−0.72 (-1.23, -0.55)	−0.04 (-0.21, 0.13)	0.64 (0.47, 0.81)	1.49 (1.15, 1.84)	<0.001
Sex					<0.001
Female	3487 (59.7%)	3205 (54.9%)	2956 (49.9%)	2626 (44.8%)	
Male	2355 (40.3%)	2629 (45.1%)	2962 (50.1%)	3240 (55.2%)	
Ethnicity					0.029
Non-white or unknown	191 (3.3%)	175 (3.0%)	176 (3.0%)	139 (2.4%)	
White	5651 (96.7%)	5659 (97.0%)	5742 (97.0%)	5727 (97.6%)	
Household income					0.005
High	2301 (39.4%)	2242 (38.4%)	2241 (37.9%)	2146 (36.6%)	
Low	1501 (25.7%)	1607 (27.5%)	1643 (27.8%)	1685 (28.7%)	
Middle	1647 (28.2%)	1621 (27.8%)	1702 (28.8%)	1678 (28.6%)	
Unknown	393 (6.7%)	364 (6.2%)	332 (5.6%)	357 (6.1%)	
Education					<0.001
College or university degree	3321 (56.8%)	3202 (54.9%)	3184 (53.8%)	3011 (51.3%)	
No formal qualification	153 (2.6%)	214 (3.7%)	208 (3.5%)	222 (3.8%)	
Other or unknown	7 (0.1%)	11 (0.2%)	8 (0.1%)	3 (0.1%)	
Secondary or post-secondary qualification	1867 (32.0%)	1908 (32.7%)	1966 (33.2%)	2132 (36.3%)	
Vocational or professional qualification	494 (8.5%)	499 (8.6%)	552 (9.3%)	498 (8.5%)	
Smoking status					<0.001
Ever or unknown	2452 (42.0%)	2367 (40.6%)	2308 (39.0%)	2203 (37.6%)	
Never	3390 (58.0%)	3467 (59.4%)	3610 (61.0%)	3663 (62.4%)	
Alcohol drinking status					0.633
Current	5591 (95.7%)	5576 (95.6%)	5637 (95.3%)	5611 (95.7%)	
Non-current or unknown	251 (4.3%)	258 (4.4%)	281 (4.7%)	255 (4.3%)	
Diabetes					0.711
No	5705 (97.7%)	5686 (97.5%)	5772 (97.5%)	5709 (97.3%)	
Yes	137 (2.3%)	148 (2.5%)	146 (2.5%)	157 (2.7%)	
Hypertension					0.500
No	1978 (33.9%)	1927 (33.0%)	1974 (33.4%)	1910 (32.6%)	
Yes	3864 (66.1%)	3907 (67.0%)	3944 (66.6%)	3956 (67.4%)	

Values are median (IQR) for continuous variables and n (%) for categorical variables. P values were derived using Kruskal-Wallis tests for continuous variables and Pearson chi-square tests for categorical variables.

Baseline characteristics differed across uPDI quartiles. Compared with Q1, the Q4 group was younger (median 54 vs. 56 years), had a lower proportion of women (44.8% vs. 59.7%), higher total energy intake (8,783 vs. 8,321 kJ/day), and slightly lower physical activity (7.37 vs. 7.50 log MET-min/week). Body mass index differed little between Q1 and Q4 (25.63 vs. 25.85 kg/m²). Apart from body mass index, most demographic and lifestyle characteristics differed significantly across uPDI quartiles (Table 1).

The food-group profiles underlying the uPDI gradient were also distinct. From Q1 to Q4, mean refined-grain intake increased from 2.11 to 2.85 servings/day, sugar-sweetened beverage intake from 0.27 to 0.42 servings/day, and sweets and desserts from 0.70 to 1.30 servings/day. In contrast, mean fruit intake decreased from 2.82 to 1.65 servings/day, vegetable intake from 3.35 to 1.93 servings/day, and whole-grain intake from 1.33 to 1.16 servings/day. Non-consumption was common for nuts and sugar-sweetened beverages; however, the expected intake gradients remained evident across quartiles (Supplementary Table S1).

hPDI and uPDI were weakly positively correlated (Pearson r = 0.230; Spearman rho = 0.230; both P < 0.001). The corresponding variance inflation factors were 1.16 for hPDI and 1.13 for uPDI, indicating no material collinearity (Supplementary Table S2).

### 3.2 Overall Associations of hPDI and uPDI with Whole-Brain IDPs

In Model 2, uPDI was associated with multiple brain IDPs, with significant associations concentrated in diffusion MRI white-matter tract metrics and structural MRI volume metrics. After within-category FDR correction, 187 diffusion MRI white-matter IDPs and 110 structural MRI volume IDPs were associated with uPDI at category-FDR < 0.05. In addition, 2 and 4 IDPs reached category-FDR < 0.05 among functional MRI/T2star metrics and other imaging phenotypes, respectively (Table 2).

**Table 2:** IDP-wide association signals for hPDI and uPDI.

Diet score	IDP category	IDPs tested	Nominal P < 0.05	Category-FDR<0.05	Global-FDR<0.05	Strongest IDP	Strongest beta	Strongest P
Healthy plant-based diet index	brain diffusion MRI / white-matter tracts	675	98	1	2	Weighted-mean MO in tract middle cerebellar peduncle	−0.028	<0.001
Healthy plant-based diet index	brain functional MRI / T2star	30	10	4	2	Median T2star in hippocampus (left)	0.027	<0.001
Healthy plant-based diet index	brain structural MRI volumes	176	40	19	11	Volume of grey matter in VIIIb Cerebellum (right)	0.026	<0.001
Healthy plant-based diet index	other imaging phenotype	20	8	4	3	Mean rfMRI head motion, averaged across space and time points	−0.056	<0.001
Unhealthy plant-based diet index	brain diffusion MRI / white-matter tracts	675	283	187	200	Mean FA in fornix on FA skeleton	0.029	<0.001
Unhealthy plant-based diet index	brain functional MRI / T2star	30	7	2	7	Median T2star in putamen (left)	0.023	<0.001
Unhealthy plant-based diet index	brain structural MRI volumes	176	119	110	104	Volume of grey matter (normalised for head size)	0.038	<0.001
Unhealthy plant-based diet index	other imaging phenotype	20	8	4	5	Inverted temporal signal-to-noise ratio in pre-processed rfMRI	−0.022	<0.001

Model 2 adjusted for age, sex, ethnicity, household income, education, smoking, alcohol drinking status, physical activity, and total energy intake. Category-FDR was calculated within each IDP category.

By comparison, hPDI was associated with fewer whole-brain IDPs. After category-FDR correction, hPDI was associated with 1, 19, 4, and 4 IDPs among diffusion MRI, structural MRI, functional MRI/T2star, and other imaging phenotypes, respectively (Table 2). The IDP-wide scan showed that uPDI-related signals were concentrated in structural MRI volume metrics and white-matter diffusion metrics, with more significant associations and a more concentrated signal distribution than hPDI (Fig. 2); hPDI also showed some FDR-significant associations, but these were fewer in number and more dispersed.

**Fig. 2:**
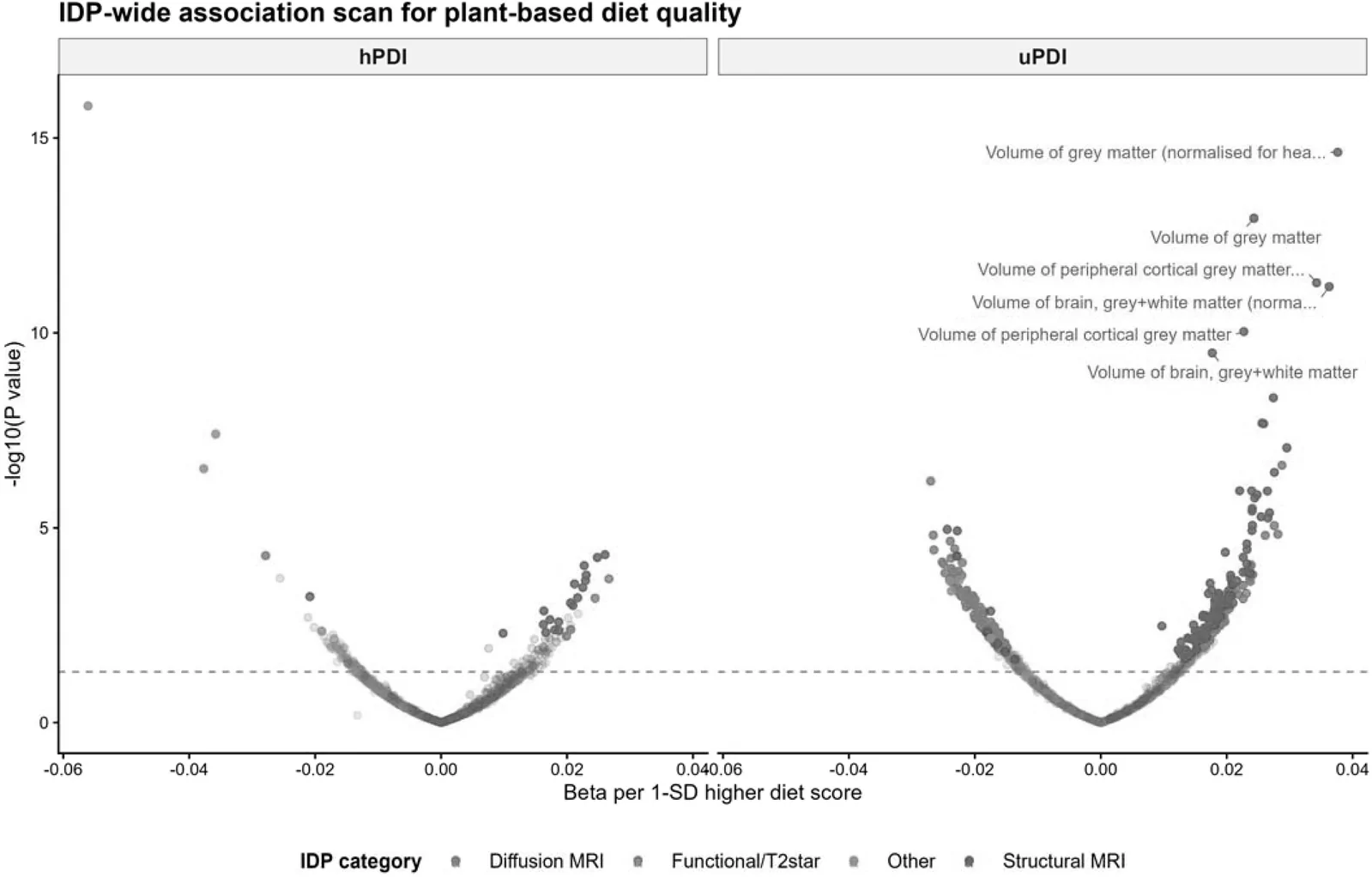
Imaging-wide associations of hPDI and uPDI with standardized brain IDPs. Each point is one IDP, colored by imaging category; highlighted points met category-level false-discovery-rate (FDR) < 0.05.

When hPDI and uPDI were included simultaneously, uPDI retained category-FDR-significant associations with 89 structural MRI volume IDPs and 46 diffusion MRI white-matter IDPs. hPDI did not retain category-FDR-significant structural or diffusion associations in the mutually adjusted models, although two functional MRI/T2star and three other imaging phenotypes remained significant (Supplementary Table S3).

### 3.3 Interpretable uPDI-Associated Imaging Phenotypes

After excluding technical or quality-control-related IDPs (image quality, head motion, scanner-table position, registration differences, and signal-to-noise ratio), uPDI remained associated with multiple structural MRI and diffusion MRI metrics with clear biological interpretation (Fig. 3). Among structural MRI metrics, uPDI was positively associated with several whole-brain or large-scale tissue-volume measures, including normalized grey-matter volume (beta = 0.038, 95% CI 0.028 to 0.047), grey-matter volume (beta = 0.024, 95% CI 0.018 to 0.031), normalized peripheral cortical grey-matter volume (beta = 0.034, 95% CI 0.025 to 0.044), and normalized whole-brain grey-plus-white-matter volume (beta = 0.036, 95% CI 0.026 to 0.047). Among subcortical structures, left and right thalamic volumes were also among the leading uPDI-associated structural signals.

**Fig. 3:**
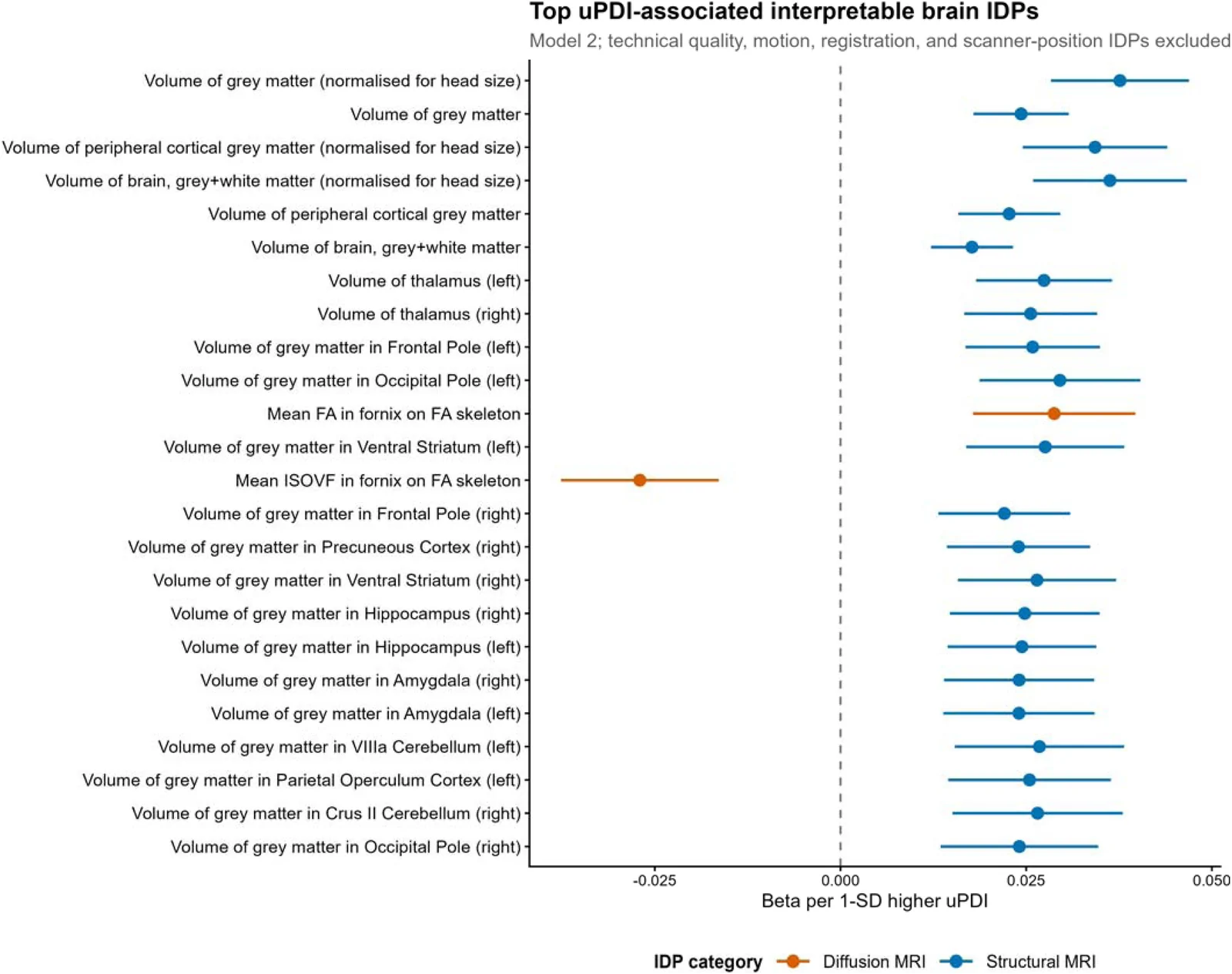
Top biologically interpretable IDPs associated with uPDI. Technical quality, head motion, registration, scanner-position, and related image-quality IDPs were excluded from this display. Points show standardized beta coefficients with 95% confidence intervals.

Among white-matter microstructure metrics, uPDI was positively associated with fornix fractional anisotropy (FA) (beta = 0.029, 95% CI 0.018 to 0.040). In addition, diffusion metrics in white-matter tracts including the fornix, corona radiata, medial lemniscus, and inferior fronto-occipital fasciculus appeared among the top interpretable uPDI-associated IDPs (Fig. 3 and Supplementary Table S4). The interpretable hPDI-associated IDPs are presented in Supplementary Table S5 and Supplementary Figure S1, and the comparison of hPDI and uPDI beta distributions across the same IDPs is shown in Supplementary Figure S2.

Overall, the associations of uPDI with brain imaging phenotypes involved both structural MRI volume metrics and white-matter microstructure metrics, forming a broad imaging-wide association pattern. Because the biological direction of different diffusion MRI metrics must be interpreted in the context of the specific metric type and white-matter tract anatomy, we report these findings as whole-brain imaging-wide association results rather than interpreting them directly as pathological changes in specific brain regions or white-matter tracts.

### 3.4 Comparison across additional dietary scores

In the supplementary diet-score analyses, the overall PDI showed category-FDR-significant associations with 176 diffusion MRI, 108 structural MRI, 11 functional MRI/T2star, and 4 other imaging phenotypes. E-DII likewise showed FDR-significant associations with multiple IDPs, with signals concentrated in structural MRI volume and white-matter tract diffusion metrics. By contrast, DII, DASH, and AHEI showed mainly nominal associations without stable FDR-significant signals (Supplementary Table S6 and Supplementary Figure S3).

In the same 23,460-participant Model 2 sample, uPDI and the overall PDI were moderately correlated (Pearson r = 0.555; Spearman rho = 0.543; both P < 0.001), with variance inflation factors of 1.56 and 1.62, respectively (Supplementary Table S8). In models containing both scores, uPDI retained category-FDR-significant associations with 20 structural and 1 diffusion IDP, whereas the overall PDI retained 35 structural, 42 diffusion, 8 functional MRI/T2star, and 5 other imaging associations (Supplementary Table S9). Complete IDP-specific results are provided in Supplementary Data S1.

These supplementary results show that dietary scores differed in their associations with brain imaging phenotypes. The broad overall PDI signal indicates that the degree of plant-based eating was related to the imaging phenome, while the weak correlation and mutually adjusted results for hPDI and uPDI demonstrate that the quality-based indices captured distinguishable information. uPDI was moderately correlated with overall PDI, and its reduced structural signal and single remaining diffusion association after simultaneous adjustment suggest that a substantial part, but not all, of the uPDI pattern overlapped with overall plant-versus-animal dietary composition. uPDI, PDI, and E-DII exhibited the most widespread imaging associations, whereas hPDI, DII, DASH, and AHEI showed comparatively fewer significant associations.

## 4 Discussion

In this imaging-wide association study of 23,460 participants, we systematically mapped healthy and unhealthy plant-based diet quality against 910 whole-brain imaging-derived phenotypes. uPDI was associated with a broad set of structural and white-matter imaging phenotypes, with 110 structural-volume and 187 diffusion white-matter IDPs surpassing category-level FDR correction, well beyond the corresponding signal for hPDI. hPDI and uPDI were only weakly correlated and had low variance inflation factors, and uPDI retained structural and diffusion associations after both indices were entered in the same model. The overall PDI and E-DII also produced widespread imaging signals, and when uPDI and the overall PDI were entered in the same model, the number of category-FDR-significant uPDI associations decreased substantially, from 110 to 20 structural IDPs and from 187 to 1 diffusion IDP. Together, these findings indicate that much of the broad uPDI signal is shared with overall plant-versus-animal dietary composition, whereas a smaller independent uPDI signal remains. The associations were modest, and their biological meaning remains uncertain.

These results extend a substantial body of evidence linking plant-based diet quality to systemic and brain health. In large prospective cohorts, higher hPDI has been associated with lower risk of coronary heart disease and type 2 diabetes, whereas higher uPDI has been associated with higher risk of both [4,5]. More recently, in UK Biobank, a healthful plant-based diet was associated with lower risk of dementia and depression, while an unhealthful plant-based diet was associated with higher risk [19]. In the present analysis, hPDI was associated with fewer than 30 of the 910 IDPs after category-level FDR correction, although several of these associations were directionally consistent with previous literature. Our imaging-wide findings provide additional evidence that plant-based dietary indices may capture heterogeneous relationships with brain imaging phenotypes. In this analysis, uPDI showed the more widespread imaging signal of the two quality-based indices, whereas hPDI demonstrated fewer associations. However, the direction of the uPDI-related structural and diffusion associations was not uniformly consistent with conventional expectations of dietary healthfulness, and these findings should therefore not be interpreted as indicating beneficial or harmful effects of a specific dietary pattern [7,8]. By scanning the whole imaging phenome rather than a small number of a priori regions, our analysis adds breadth that single-region or single-score studies cannot provide.

Interpretation of the uPDI findings also depends on their overlap with the overall PDI. uPDI and the overall PDI share the reverse scoring of animal foods and the positive scoring of less-healthy plant foods and differ only in the scoring direction of healthy plant foods; consistent with this shared structure, the two scores were moderately correlated (Pearson r = 0.555). When both scores were entered in the same model, the number of category-FDR-significant uPDI associations decreased from 110 to 20 for structural IDPs and from 187 to 1 for diffusion IDPs, whereas the overall PDI retained 35 structural and 42 diffusion associations. The strongest remaining structural association of uPDI was with normalized grey-matter volume (beta = 0.029, compared with 0.038 in the model without the overall PDI). A substantial proportion of the imaging associations initially observed for uPDI therefore overlapped with the general plant-versus-animal composition of the diet captured by the overall PDI. The 20 structural and 1 diffusion associations that persisted indicate that uPDI retained an independent signal beyond overall plant-based dietary composition, but this signal was considerably more limited than that observed for uPDI alone. Accordingly, the broad imaging pattern observed for uPDI should not be interpreted as reflecting specifically the nutritional quality of plant-based foods, and only the smaller residual signal may relate to the distinction between healthy and less-healthy plant foods.

The uPDI-associated diffusion signal was concentrated in white-matter tracts, including the fornix, corona radiata, medial lemniscus, and inferior fronto-occipital fasciculus. Fractional anisotropy and related diffusion metrics index the orientational coherence and microstructural organization of white matter but are not specific to any single biological process, since factors such as fiber density, myelination, axonal geometry, and crossing-fiber configuration all influence them [23]. Large analyses have shown that white-matter microstructure is strongly patterned by age and becomes increasingly correlated across tracts in later life [24]. This age-related patterning underscores the importance of accounting for age when interpreting diet–imaging associations, as was done in our primary models. Because diffusion abnormalities and white-matter hyperintensities are central features of cerebral small-vessel disease [25,26], and because midlife systemic inflammation has been linked prospectively to later white-matter microstructural change [27], a diet-related white-matter signature is biologically plausible. The microbiota-gut-brain axis may provide an additional pathway linking dietary composition to brain phenotypes through interactions among microbial metabolites, immune signaling, and neuroendocrine pathways [28]. Nonetheless, the direction of individual diffusion associations cannot be interpreted as uniformly beneficial or harmful, and we therefore report them as imaging-wide associations rather than as localized pathology. In addition, only one diffusion association remained significant after adjustment for the overall PDI, indicating that this white-matter pattern was largely shared with overall plant-versus-animal dietary composition.

For structural metrics, higher uPDI was associated with greater, rather than smaller, normalized grey-matter and whole-brain tissue volumes, a direction opposite to our a priori expectation and to the smaller hippocampal and total brain volumes reported for unhealthy or "Western" dietary patterns and to the larger volumes reported for the Mediterranean diet in older cohorts [[12], [13], [14]]. Several factors may contribute to this apparently paradoxical direction. Participants in the highest uPDI quartile were younger and reported higher total energy intake, although age was explicitly accounted for in the primary multivariable model and the age difference across the extreme uPDI quartiles was relatively modest. Therefore, age-related confounding is unlikely to be a major explanation for the observed volumetric associations. However, because age is closely correlated with multiple biological, lifestyle, and health-related characteristics, the possibility that some age-associated factors were incompletely captured cannot be entirely excluded. Other unmeasured or incompletely measured characteristics may nevertheless contribute to the observed associations. Given the cross-sectional design, the temporal direction of these associations cannot be established. Reverse causation therefore remains possible, as pre-existing differences in brain structure, cognitive function, or related lifestyle behaviors may influence dietary preferences and food choices rather than the reverse. Consequently, individuals with different underlying brain-related characteristics may adopt different dietary patterns over time, resulting in associations that do not necessarily reflect a direct effect of diet on brain structure. Accordingly, the structural associations should be read as robust, FDR-controlled statistical signals at the imaging-phenome level rather than as evidence that unhealthy plant-based diets enlarge the brain.

The overlap between the uPDI and energy-adjusted Dietary Inflammatory Index signals provides a descriptive comparison of dietary association patterns. Both scores up-weight refined and pro-inflammatory foods, and, apart from the overall PDI, the E-DII was the only comparison score to show a substantial, although less widespread, imaging signal. However, the convergence of uPDI and E-DII associations should not be interpreted as evidence that inflammatory mechanisms account for the observed imaging pattern. In particular, the positive associations of uPDI with several volume measures and white-matter metrics do not conform to the expected direction of a simple model in which a pro-inflammatory diet leads to adverse brain structural changes. The observed overlap may instead indicate that uPDI and E-DII capture some shared dietary characteristics, while the specific biological processes underlying the imaging associations remain uncertain. High intakes of sugar-sweetened beverages and low intakes of whole grains, both characteristic of a high-uPDI pattern, have each been independently linked to adverse brain and cardiometabolic outcomes [7,8]. Thus, the similarity between the uPDI and E-DII patterns should be regarded as an empirical association rather than mechanistic evidence, particularly given the cross-sectional design and the unexpected direction of several structural associations.

From a public-health perspective, these findings highlight that different plant-based dietary profiles may exhibit distinct relationships with brain imaging phenotypes. Diet is one of several modifiable contributors to brain health and dementia risk across the life course [6], and lifestyle factors such as physical activity can measurably alter brain structure, including hippocampal volume, in older adults [29]. Because population dietary guidance increasingly promotes plant-forward and sustainable eating patterns [2], future studies should examine whether the quality of plant foods is related to brain health independently of overall plant-based dietary composition, for example by analyzing quality-based and overall plant-based indices in the same models. Imaging-derived phenotypes may serve as intermediate markers for examining these relationships in future dietary studies and trials.

This study has several strengths. We drew on one of the largest available population neuroimaging resources, combining harmonized 24 -h dietary recall data with brain MRI in more than twenty thousand participants; at this scale, brain-phenotype associations become more reproducible and effect-size inflation is reduced [15]. Rather than testing a small number of pre-selected regions, we adopted an imaging-wide, hypothesis-agnostic design across 910 IDPs with both category-level and global FDR control. We deliberately separated biologically interpretable phenotypes from technical and quality-control metrics, adjusted structural analyses for head size using T1 volumetric scaling, evaluated correlation, collinearity, and mutual adjustment of hPDI and uPDI, and benchmarked the quality-based indices against the overall PDI and four additional diet-quality and inflammatory scores. The dietary instrument and imaging pipeline are both validated and widely used [10,21].

Several limitations should also be considered. The analysis was cross-sectional, so temporal and causal relationships between diet and brain structure cannot be established. Dietary exposure was derived from self-reported 24 -h recalls, which are subject to measurement error and within-person variability that generally bias associations toward the null. Furthermore, because dietary scores and imaging phenotypes were standardized, effect estimates facilitate comparisons across different imaging measures but cannot be directly translated into absolute changes in dietary intake or brain measurements. Despite extensive covariate adjustment, unmeasured or incompletely measured confounding by factors related to body composition, lifestyle, and dietary behavior may remain. Although age was included in the primary model, the cross-sectional nature of the analysis means that complex age-related differences cannot be fully separated from other correlated characteristics. These considerations warrant caution in interpreting the counterintuitive direction of the structural volume associations. Participants are healthier and less socioeconomically deprived than the general population, reflecting a "healthy-volunteer" selection that may limit generalizability even when internal exposure-outcome associations remain valid [30]. The biological direction of many diffusion and volumetric associations is intrinsically ambiguous and cannot be mapped onto specific pathological processes without targeted follow-up. Finally, the imaging and dietary data were predominantly from European-ancestry participants, and replication in independent and more diverse cohorts, ideally with longitudinal imaging, will be needed to confirm these imaging-wide associations.

The construction of the dietary scores introduces an additional limitation. For whole grains, legumes, refined grains, potatoes, poultry, and dairy, unreported intake was treated as missing rather than as zero intake, and the missing component was omitted from the index sum. Because the Oxford WebQ is a 24 -h recall, some unreported intakes may represent non-consumption on the recall day. Deriving quintile cut points only among participants with recorded intake may therefore alter the component distributions. In addition, several healthy plant-food components occupied only part of the 1–5 scoring range, reducing their potential contribution to variation in uPDI. Together, these features may make uPDI more similar to the overall PDI, which shares the scoring of animal foods and less-healthy plant foods. They may contribute to the attenuation after mutual adjustment and could underestimate associations specific to plant-food quality. The direction and magnitude of any resulting bias remain uncertain. Future work should distinguish non-consumption from missing responses, assign zero intake to confirmed non-consumers, include all components in the index, and examine whether the attenuation persists.

## 5 Conclusion

In a large imaging-wide association study in UK Biobank, unhealthy plant-based diet quality was associated with broad structural and white-matter imaging signatures across the brain and remained associated with multiple IDPs after mutual adjustment for healthy plant-based diet quality. Notably, higher uPDI was associated with greater normalized grey-matter volume and higher fornix fractional anisotropy, a direction that was contrary to expectations based on prior literature. The overall PDI and E-DII also showed widespread imaging associations. After mutual adjustment for the overall PDI, however, the number of category-FDR-significant uPDI associations decreased substantially, from 110 to 20 structural IDPs and from 187 to 1 diffusion IDP, indicating that the widespread associations observed for uPDI alone largely overlapped with overall plant-versus-animal dietary composition and that only a limited part of this pattern was independent of the overall PDI. Accordingly, the observed imaging associations cannot be attributed specifically to the nutritional quality of plant-based foods. Longitudinal and multi-ancestry studies with repeated imaging are required to clarify the directionality and biological meaning of these associations.

## Authorship contribution statement

Concept and design: Boding Wang; Acquisition of data: Kai Wang; Statistical analysis: Liyuan Han; Interpretation of data: Chuyi Cai, Feiyu Chen; Drafting of the manuscript: Boding Wang, Kai Wang, Liyuan Han; Critical revision of the manuscript for important intellectual content: Yiling Wang, Chuyi Cai, Feiyu Chen; All authors read and approved the final manuscript.

## Ethics statement

Ethical approval was obtained from the North West Multi-centre Research Ethics Committee in the UK. All participants provided informed consent at enrolment. The current analyses were conducted under application 78619.

## Declaration of Generative AI and AI-assisted technologies in the writing process

During this revision, ChatGPT (OpenAI) was used for English language editing and assistance with the wording of the manuscript and response letter. No study data, statistical results, figures, images, or artwork were generated or modified using generative AI in this revision. The authors take responsibility for the final content.

## Funding sources

This work was supported by the Project of NINGBO Leading Medical & Health Discipline [grant number 2026-A24] , 2025 Zhejiang Province “Pioneer” and “Leading Goose” Science and Technology Plan Project [grant number 2025C02252(SD2)], Zhu Xiu Shan Talent Project of Ningbo No.2 Hospital [grant number 2023HMJQ-19], 2023 Ningbo Youth Science and Technology Innovation Leading Talent Project [grant number 2024QL024], The first level of the 2023 Ningbo Yongjiang Talent Programme Leading Elite Talent Training Project [grant number 2024RC001] and Supporting high-quality development to cultivate a small but strong clinical innovation team for acute large-vessel occlusive cerebral infarction [grant number 2026-XEQ-01ZY].

## Data statement

The datasets generated and analyzed during the current study are available from the corresponding author on reasonable request.

## Clinical trial

Not applicable.

## Declaration of competing interest

The authors declare that they have no known competing financial interests or personal relationships that could have appeared to influence the work reported in this paper. The authors declare the following financial interests/personal relationships which may be considered as potential competing interests:
