# Supplementary material for: Healthy and unhealthy plant-based diet quality and whole-brain imaging-derived phenotypes: an imaging-wide association study
Source: J Nutr Health Aging. 2026 Nov 3;31(1):101027. doi: 10.1016/j.jnha.2026.101027 (PMC13756244; doi:10.1016/j.jnha.2026.101027)
Supplement: Supplementary file 1 [file mmc1.docx]

**Supplementary Material**

Supplementary tables and figures provide full signal summaries, top interpretable IDP results for hPDI and uPDI, comparison across additional diet scores, component-specific scoring-group distributions, and uPDI–PDI correlation and mutually adjusted analyses. Complete IDP-specific results for the uPDI–PDI mutually adjusted analysis are provided in Supplementary Data S1.

**Supplementary Table S1. Food-group intakes across uPDI quartiles**

| **Food group** | **Scoring domain** | **Q1, mean (SD), servings/day** | **Q1, non-consumers, %** | **Q2, mean (SD), servings/day** | **Q2, non-consumers, %** | **Q3, mean (SD), servings/day** | **Q3, non-consumers, %** | **Q4, mean (SD), servings/day** | **Q4, non-consumers, %** | **P value for intake** | **P value for non-consumer prevalence** |
| --- | --- | --- | --- | --- | --- | --- | --- | --- | --- | --- | --- |
| Whole grains | Healthy plant foods | 1.33 (0.62) | 0.0 | 1.27 (0.60) | 0.0 | 1.24 (0.59) | 0.0 | 1.16 (0.53) | 0.0 | <0.001 | <0.001 |
| Fruits | Healthy plant foods | 2.82 (1.76) | 3.8 | 2.37 (1.58) | 5.0 | 2.09 (1.41) | 5.2 | 1.66 (1.20) | 6.8 | <0.001 | <0.001 |
| Vegetables | Healthy plant foods | 3.36 (2.15) | 2.4 | 2.78 (1.88) | 3.4 | 2.41 (1.67) | 3.4 | 1.94 (1.34) | 3.4 | <0.001 | 0.002 |
| Nuts | Healthy plant foods | 0.20 (0.36) | 63.3 | 0.16 (0.33) | 67.1 | 0.14 (0.32) | 70.2 | 0.11 (0.26) | 75.0 | <0.001 | <0.001 |
| Legumes | Healthy plant foods | 1.00 (0.57) | 0.0 | 0.95 (0.53) | 0.0 | 0.91 (0.48) | 0.0 | 0.83 (0.46) | 0.0 | <0.001 | <0.001 |
| Refined grains | Less-healthy plant foods | 2.12 (0.97) | 0.0 | 2.40 (1.08) | 0.0 | 2.57 (1.09) | 0.0 | 2.85 (1.10) | 0.0 | <0.001 | <0.001 |
| Potatoes | Less-healthy plant foods | 1.27 (0.71) | 0.0 | 1.37 (0.76) | 0.0 | 1.41 (0.77) | 0.0 | 1.52 (0.80) | 0.0 | <0.001 | <0.001 |
| Sugar-sweetened beverages | Less-healthy plant foods | 0.28 (0.52) | 60.4 | 0.32 (0.56) | 54.8 | 0.36 (0.57) | 50.7 | 0.42 (0.64) | 46.2 | <0.001 | <0.001 |
| Sweets and desserts | Less-healthy plant foods | 0.71 (0.79) | 25.2 | 0.92 (0.90) | 17.2 | 1.06 (0.97) | 14.0 | 1.31 (1.02) | 8.4 | <0.001 | <0.001 |
| Red meat | Animal foods | 0.37 (0.39) | 39.4 | 0.32 (0.35) | 41.9 | 0.29 (0.32) | 41.3 | 0.27 (0.28) | 38.2 | <0.001 | <0.001 |
| Processed meat | Animal foods | 0.60 (0.76) | 42.9 | 0.54 (0.71) | 44.0 | 0.51 (0.67) | 42.0 | 0.48 (0.63) | 40.3 | <0.001 | <0.001 |
| Poultry | Animal foods | 1.16 (0.60) | 0.0 | 1.07 (0.48) | 0.0 | 1.08 (0.50) | 0.0 | 1.05 (0.48) | 0.0 | <0.001 | <0.001 |
| Fish | Animal foods | 0.45 (0.48) | 34.9 | 0.35 (0.40) | 39.6 | 0.30 (0.36) | 42.7 | 0.24 (0.32) | 46.4 | <0.001 | <0.001 |
| Eggs | Animal foods | 0.34 (0.46) | 50.8 | 0.28 (0.41) | 53.8 | 0.27 (0.38) | 53.3 | 0.24 (0.35) | 53.6 | <0.001 | 0.004 |
| Dairy | Animal foods | 2.13 (1.14) | 0.0 | 1.95 (1.11) | 0.0 | 1.81 (1.07) | 0.0 | 1.60 (0.97) | 0.0 | <0.001 | <0.001 |

Values are mean (SD) in servings/day or the percentage of non-consumers. Quartiles were defined in the final hPDI/uPDI Model 2 analytic sample.

**Supplementary Table S2. Correlation and collinearity diagnostics for hPDI and uPDI**

| **Analysis** | **Participants** | **Estimate** | **P value** | **Definition** |
| --- | --- | --- | --- | --- |
| Pearson correlation: hPDI and uPDI | 23460 | 0.230 | <0.001 | Linear correlation between standardized scores |
| Spearman correlation: hPDI and uPDI | 23460 | 0.230 | <0.001 | Rank correlation between standardized scores |
| Variance inflation factor: hPDI | 23460 | 1.158 |  | Calculated against uPDI and Model 2 covariates |
| Variance inflation factor: uPDI | 23460 | 1.127 |  | Calculated against hPDI and Model 2 covariates |

Variance inflation factors were calculated from models containing both plant-based diet indices and the Model 2 covariates.

**Supplementary Table S3. Mutually adjusted hPDI and uPDI imaging-wide association summary**

| **Diet score** | **IDP category** | **Number of IDPs** | **Nominal P <0.05** | **Category FDR <0.05** | **Global FDR <0.05** | **Strongest associated IDP** | **Beta** | **P value** |
| --- | --- | --- | --- | --- | --- | --- | --- | --- |
| Healthy plant-based diet index | other imaging phenotype | 20 | 4 | 3 | 3 | Mean rfMRI head motion, averaged across space and time points | -0.057 | <0.001 |
| Unhealthy plant-based diet index | other imaging phenotype | 20 | 5 | 0 | 0 | Intensity scaling for dMRI | -0.020 | 0.009 |
| Healthy plant-based diet index | brain structural MRI volumes | 176 | 16 | 0 | 0 | Volume of grey matter in VIIIb Cerebellum (right) | 0.025 | <0.001 |
| Unhealthy plant-based diet index | brain structural MRI volumes | 176 | 100 | 89 | 62 | Volume of grey matter (normalised for head size) | 0.038 | <0.001 |
| Healthy plant-based diet index | brain functional MRI / T2star | 30 | 9 | 2 | 0 | Median T2star in hippocampus (left) | 0.025 | 0.002 |
| Unhealthy plant-based diet index | brain functional MRI / T2star | 30 | 3 | 0 | 0 | Median T2star in putamen (left) | 0.020 | 0.008 |
| Healthy plant-based diet index | brain diffusion MRI / white-matter tracts | 675 | 51 | 0 | 0 | Weighted-mean OD in tract forceps major | -0.028 | <0.001 |
| Unhealthy plant-based diet index | brain diffusion MRI / white-matter tracts | 675 | 201 | 46 | 74 | Mean MO in external capsule on FA skeleton (left) | 0.031 | <0.001 |

Each model included hPDI and uPDI simultaneously and adjusted for the Model 2 covariates; structural volume models additionally included T1 volumetric scaling where applicable.

**Supplementary Table S4. Top uPDI-associated interpretable IDPs**

| **IDP** | **IDP category** | **Beta (95% CI)** | **P value** | **Category FDR** | **Global FDR** |
| --- | --- | --- | --- | --- | --- |
| Volume of grey matter (normalised for head size) | brain structural MRI volumes | 0.038 (0.028, 0.047) | <0.001 | <0.001 | <0.001 |
| Volume of grey matter | brain structural MRI volumes | 0.024 (0.018, 0.031) | <0.001 | <0.001 | <0.001 |
| Volume of peripheral cortical grey matter (normalised for head size) | brain structural MRI volumes | 0.034 (0.025, 0.044) | <0.001 | <0.001 | <0.001 |
| Volume of brain, grey+white matter (normalised for head size) | brain structural MRI volumes | 0.036 (0.026, 0.047) | <0.001 | <0.001 | <0.001 |
| Volume of peripheral cortical grey matter | brain structural MRI volumes | 0.023 (0.016, 0.030) | <0.001 | <0.001 | <0.001 |
| Volume of brain, grey+white matter | brain structural MRI volumes | 0.018 (0.012, 0.023) | <0.001 | <0.001 | <0.001 |
| Volume of thalamus (left) | brain structural MRI volumes | 0.027 (0.018, 0.037) | <0.001 | <0.001 | <0.001 |
| Volume of thalamus (right) | brain structural MRI volumes | 0.026 (0.017, 0.035) | <0.001 | <0.001 | <0.001 |
| Volume of grey matter in Frontal Pole (left) | brain structural MRI volumes | 0.026 (0.017, 0.035) | <0.001 | <0.001 | <0.001 |
| Volume of grey matter in Occipital Pole (left) | brain structural MRI volumes | 0.030 (0.019, 0.040) | <0.001 | <0.001 | <0.001 |
| Mean FA in fornix on FA skeleton | brain diffusion MRI / white-matter tracts | 0.029 (0.018, 0.040) | <0.001 | <0.001 | <0.001 |
| Volume of grey matter in Ventral Striatum (left) | brain structural MRI volumes | 0.028 (0.017, 0.038) | <0.001 | <0.001 | <0.001 |
| Mean ISOVF in fornix on FA skeleton | brain diffusion MRI / white-matter tracts | -0.027 (-0.038, -0.016) | <0.001 | <0.001 | <0.001 |
| Volume of grey matter in Frontal Pole (right) | brain structural MRI volumes | 0.022 (0.013, 0.031) | <0.001 | <0.001 | <0.001 |
| Volume of grey matter in Precuneous Cortex (right) | brain structural MRI volumes | 0.024 (0.014, 0.034) | <0.001 | <0.001 | <0.001 |
| Volume of grey matter in Ventral Striatum (right) | brain structural MRI volumes | 0.026 (0.016, 0.037) | <0.001 | <0.001 | <0.001 |
| Volume of grey matter in Hippocampus (right) | brain structural MRI volumes | 0.025 (0.015, 0.035) | <0.001 | <0.001 | <0.001 |
| Volume of grey matter in Hippocampus (left) | brain structural MRI volumes | 0.024 (0.014, 0.034) | <0.001 | <0.001 | <0.001 |
| Volume of grey matter in Amygdala (right) | brain structural MRI volumes | 0.024 (0.014, 0.034) | <0.001 | <0.001 | <0.001 |
| Volume of grey matter in Amygdala (left) | brain structural MRI volumes | 0.024 (0.014, 0.034) | <0.001 | <0.001 | <0.001 |
| Volume of grey matter in VIIIa Cerebellum (left) | brain structural MRI volumes | 0.027 (0.015, 0.038) | <0.001 | <0.001 | <0.001 |
| Volume of grey matter in Parietal Operculum Cortex (left) | brain structural MRI volumes | 0.025 (0.015, 0.036) | <0.001 | <0.001 | <0.001 |
| Volume of grey matter in Crus II Cerebellum (right) | brain structural MRI volumes | 0.027 (0.015, 0.038) | <0.001 | <0.001 | <0.001 |
| Volume of grey matter in Occipital Pole (right) | brain structural MRI volumes | 0.024 (0.013, 0.035) | <0.001 | <0.001 | <0.001 |
| Mean MO in external capsule on FA skeleton (left) | brain diffusion MRI / white-matter tracts | 0.028 (0.015, 0.040) | <0.001 | 0.002 | <0.001 |

Supplementary Table S4 lists the leading non-technical uPDI-associated IDPs after excluding image-quality, motion, registration, and scanner-position phenotypes.

**Supplementary Table S5. Top hPDI-associated interpretable IDPs**

| **IDP** | **IDP category** | **Beta (95% CI)** | **P value** | **Category FDR** | **Global FDR** |
| --- | --- | --- | --- | --- | --- |
| Volume of grey matter in VIIIb Cerebellum (right) | brain structural MRI volumes | 0.026 (0.013, 0.039) | <0.001 | 0.005 | 0.009 |
| Weighted-mean MO in tract middle cerebellar peduncle | brain diffusion MRI / white-matter tracts | -0.028 (-0.041, -0.014) | <0.001 | 0.035 | 0.009 |
| Volume of grey matter in V Cerebellum (left) | brain structural MRI volumes | 0.025 (0.013, 0.037) | <0.001 | 0.005 | 0.009 |
| Volume of grey matter in Ventral Striatum (left) | brain structural MRI volumes | 0.023 (0.011, 0.034) | <0.001 | 0.005 | 0.012 |
| Volume of grey matter in V Cerebellum (right) | brain structural MRI volumes | 0.023 (0.011, 0.035) | <0.001 | 0.007 | 0.018 |
| Median T2star in hippocampus (left) | brain functional MRI / T2star | 0.027 (0.013, 0.041) | <0.001 | 0.006 | 0.019 |
| Volume of hippocampus (left) | brain structural MRI volumes | 0.023 (0.011, 0.035) | <0.001 | 0.008 | 0.019 |
| Volume of grey matter in Ventral Striatum (right) | brain structural MRI volumes | 0.021 (0.010, 0.033) | <0.001 | 0.008 | 0.021 |
| Volume of grey matter in VIIIa Cerebellum (right) | brain structural MRI volumes | 0.022 (0.010, 0.035) | <0.001 | 0.009 | 0.024 |
| Volume of grey matter in I-IV Cerebellum (left) | brain structural MRI volumes | 0.022 (0.009, 0.034) | <0.001 | 0.012 | 0.036 |
| Median T2star in hippocampus (right) | brain functional MRI / T2star | 0.024 (0.010, 0.038) | <0.001 | 0.010 | 0.036 |
| Volume of accumbens (left) | brain structural MRI volumes | 0.021 (0.008, 0.033) | <0.001 | 0.015 | 0.045 |
| Volume of grey matter in VIIIb Cerebellum (left) | brain structural MRI volumes | 0.021 (0.008, 0.033) | <0.001 | 0.016 | 0.049 |
| Volume of grey matter (normalised for head size) | brain structural MRI volumes | 0.016 (0.006, 0.026) | 0.001 | 0.020 | 0.064 |
| Volume of brain, grey+white matter (normalised for head size) | brain structural MRI volumes | 0.017 (0.006, 0.028) | 0.002 | 0.031 | 0.089 |
| Volume of accumbens (right) | brain structural MRI volumes | 0.019 (0.007, 0.031) | 0.003 | 0.033 | 0.098 |
| Volume of grey matter in Hippocampus (left) | brain structural MRI volumes | 0.016 (0.006, 0.027) | 0.003 | 0.035 | 0.109 |
| Median T2star in amygdala (right) | brain functional MRI / T2star | 0.021 (0.007, 0.035) | 0.004 | 0.041 | 0.129 |
| Volume of grey matter in I-IV Cerebellum (right) | brain structural MRI volumes | 0.018 (0.006, 0.030) | 0.004 | 0.045 | 0.129 |
| Volume of grey matter in IX Cerebellum (right) | brain structural MRI volumes | 0.019 (0.006, 0.032) | 0.004 | 0.045 | 0.130 |
| Volume of grey matter in VI Cerebellum (right) | brain structural MRI volumes | 0.017 (0.005, 0.028) | 0.005 | 0.047 | 0.131 |
| Volume of grey matter | brain structural MRI volumes | 0.010 (0.003, 0.017) | 0.005 | 0.047 | 0.135 |
| Median T2star in pallidum (right) | brain functional MRI / T2star | 0.020 (0.006, 0.034) | 0.006 | 0.045 | 0.146 |

Supplementary Table S5 lists the leading non-technical hPDI-associated IDPs.

**Supplementary Table S6. Signal summary across all diet scores**

| **Score** | **Description** | **Imaging category** | **IDPs tested** | **Nominal P <0.05** | **Category FDR <0.05** | **Global FDR <0.05** | **Median beta** |
| --- | --- | --- | --- | --- | --- | --- | --- |
| AHEI | Adapted AHEI-2010 score | Diffusion mri / white-matter tracts | 675 | 22 | 0 | 0 | -0.003 |
| AHEI | Adapted AHEI-2010 score | Functional mri / t2star | 30 | 0 | 0 | 0 | 0.001 |
| AHEI | Adapted AHEI-2010 score | Structural mri volumes | 176 | 5 | 0 | 0 | -0.002 |
| AHEI | Adapted AHEI-2010 score | Other imaging phenotype | 19 | 1 | 0 | 0 | -0.005 |
| DASH | DASH score | Diffusion mri / white-matter tracts | 675 | 19 | 0 | 0 | -0.002 |
| DASH | DASH score | Functional mri / t2star | 30 | 2 | 0 | 0 | -0.020 |
| DASH | DASH score | Structural mri volumes | 176 | 7 | 0 | 0 | -0.001 |
| DASH | DASH score | Other imaging phenotype | 19 | 2 | 0 | 0 | -0.000 |
| DII | Dietary Inflammatory Index | Diffusion mri / white-matter tracts | 675 | 36 | 0 | 0 | -0.001 |
| DII | Dietary Inflammatory Index | Functional mri / t2star | 30 | 1 | 0 | 0 | -0.017 |
| DII | Dietary Inflammatory Index | Structural mri volumes | 176 | 10 | 0 | 0 | -0.003 |
| DII | Dietary Inflammatory Index | Other imaging phenotype | 19 | 2 | 0 | 0 | 0.002 |
| E-DII | Energy-adjusted Dietary Inflammatory Index | Diffusion mri / white-matter tracts | 675 | 202 | 61 | 63 | -0.002 |
| E-DII | Energy-adjusted Dietary Inflammatory Index | Functional mri / t2star | 30 | 4 | 0 | 0 | -0.007 |
| E-DII | Energy-adjusted Dietary Inflammatory Index | Structural mri volumes | 176 | 63 | 36 | 33 | 0.016 |
| E-DII | Energy-adjusted Dietary Inflammatory Index | Other imaging phenotype | 19 | 3 | 0 | 1 | -0.004 |
| hPDI | Healthy Plant-based Diet Index | Diffusion mri / white-matter tracts | 675 | 98 | 1 | 2 | -0.003 |
| hPDI | Healthy Plant-based Diet Index | Functional mri / t2star | 30 | 10 | 4 | 2 | 0.008 |
| hPDI | Healthy Plant-based Diet Index | Structural mri volumes | 176 | 40 | 19 | 11 | 0.006 |
| hPDI | Healthy Plant-based Diet Index | Other imaging phenotype | 20 | 8 | 4 | 3 | -0.013 |
| uPDI | Unhealthy Plant-based Diet Index | Diffusion mri / white-matter tracts | 675 | 283 | 187 | 200 | -0.003 |
| uPDI | Unhealthy Plant-based Diet Index | Functional mri / t2star | 30 | 7 | 2 | 7 | 0.001 |
| uPDI | Unhealthy Plant-based Diet Index | Structural mri volumes | 176 | 119 | 110 | 104 | 0.014 |
| uPDI | Unhealthy Plant-based Diet Index | Other imaging phenotype | 20 | 8 | 4 | 5 | -0.012 |
| PDI | Overall plant-based diet index | Other imaging phenotype | 20 | 6 | 4 | 5 | -0.012 |
| PDI | Overall plant-based diet index | Structural mri volumes | 176 | 115 | 108 | 93 | 0.016 |
| PDI | Overall plant-based diet index | Functional mri / t2star | 30 | 12 | 11 | 11 | 0.010 |
| PDI | Overall plant-based diet index | Diffusion mri / white-matter tracts | 675 | 295 | 176 | 195 | -0.007 |

Supplementary Table S6 compares DII, E-DII, DASH, AHEI, hPDI and uPDI across IDP categories.

**Supplementary Table S7. Food-group intake distributions and non-consumer placement across component-specific scoring categories**

| Food group | Scoring domain | PDI / hPDI / uPDI component score | n | Median (IQR), servings/day | Non-consumers within category, % | Share of all non-consumers, % |
| --- | --- | --- | --- | --- | --- | --- |
| Whole grains | Healthy plant foods | 1 / 1 / 5 | 10039 | 1.00 (1.00–1.00) | 0.0 | — |
| Whole grains | Healthy plant foods | 2 / 2 / 4 | 1127 | 1.50 (1.38–1.50) | 0.0 | — |
| Whole grains | Healthy plant foods | 3 / 3 / 3 | 3077 | 2.00 (2.00–2.00) | 0.0 | — |
| Whole grains | Healthy plant foods | 4 / 4 / 2 | — | Not formed | — | — |
| Whole grains | Healthy plant foods | 5 / 5 / 1 | — | Not formed | — | — |
| Fruits | Healthy plant foods | 1 / 1 / 5 | 4287 | 0.50 (0.00–0.67) | 28.4 | 100.0 |
| Fruits | Healthy plant foods | 2 / 2 / 4 | 4790 | 1.25 (1.00–1.50) | 0.0 | 0.0 |
| Fruits | Healthy plant foods | 3 / 3 / 3 | 5070 | 2.00 (1.75–2.17) | 0.0 | 0.0 |
| Fruits | Healthy plant foods | 4 / 4 / 2 | 5445 | 3.00 (2.62–3.25) | 0.0 | 0.0 |
| Fruits | Healthy plant foods | 5 / 5 / 1 | 3868 | 4.50 (4.00–5.38) | 0.0 | 0.0 |
| Vegetables | Healthy plant foods | 1 / 1 / 5 | 3509 | 0.50 (0.19–0.75) | 21.2 | 100.0 |
| Vegetables | Healthy plant foods | 2 / 2 / 4 | 5305 | 1.38 (1.17–1.58) | 0.0 | 0.0 |
| Vegetables | Healthy plant foods | 3 / 3 / 3 | 5806 | 2.25 (2.00–2.50) | 0.0 | 0.0 |
| Vegetables | Healthy plant foods | 4 / 4 / 2 | 4659 | 3.33 (3.00–3.67) | 0.0 | 0.0 |
| Vegetables | Healthy plant foods | 5 / 5 / 1 | 4181 | 5.17 (4.50–6.25) | 0.0 | 0.0 |
| Nuts | Healthy plant foods | 1 / 1 / 5 | 17710 | 0.00 (0.00–0.00) | 91.3 | 100.0 |
| Nuts | Healthy plant foods | 2 / 2 / 4 | 5750 | 0.50 (0.33–0.75) | 0.0 | 0.0 |
| Nuts | Healthy plant foods | 3 / 3 / 3 | — | Not formed | — | — |
| Nuts | Healthy plant foods | 4 / 4 / 2 | — | Not formed | — | — |
| Nuts | Healthy plant foods | 5 / 5 / 1 | — | Not formed | — | — |
| Legumes | Healthy plant foods | 1 / 1 / 5 | 2983 | 0.50 (0.25–0.50) | 0.0 | — |
| Legumes | Healthy plant foods | 2 / 2 / 4 | 4571 | 1.00 (1.00–1.00) | 0.0 | — |
| Legumes | Healthy plant foods | 3 / 3 / 3 | 1239 | 2.00 (1.50–2.00) | 0.0 | — |
| Legumes | Healthy plant foods | 4 / 4 / 2 | — | Not formed | — | — |
| Legumes | Healthy plant foods | 5 / 5 / 1 | — | Not formed | — | — |
| Refined grains | Less-healthy plant foods | 1 / 5 / 1 | 4085 | 1.00 (1.00–1.50) | 0.0 | — |
| Refined grains | Less-healthy plant foods | 2 / 4 / 2 | 6153 | 2.00 (2.00–2.00) | 0.0 | — |
| Refined grains | Less-healthy plant foods | 3 / 3 / 3 | 2333 | 2.50 (2.33–2.50) | 0.0 | — |
| Refined grains | Less-healthy plant foods | 4 / 2 / 4 | 3999 | 3.00 (3.00–3.00) | 0.0 | — |
| Refined grains | Less-healthy plant foods | 5 / 1 / 5 | 4360 | 4.00 (3.50–4.50) | 0.0 | — |
| Potatoes | Less-healthy plant foods | 1 / 5 / 1 | 10684 | 1.00 (0.75–1.00) | 0.0 | — |
| Potatoes | Less-healthy plant foods | 2 / 4 / 2 | 6575 | 2.00 (1.50–2.00) | 0.0 | — |
| Potatoes | Less-healthy plant foods | 3 / 3 / 3 | 1975 | 3.00 (2.50–3.00) | 0.0 | — |
| Potatoes | Less-healthy plant foods | 4 / 2 / 4 | — | Not formed | — | — |
| Potatoes | Less-healthy plant foods | 5 / 1 / 5 | — | Not formed | — | — |
| Sugar-sweetened beverages | Less-healthy plant foods | 1 / 5 / 1 | 19622 | 0.00 (0.00–0.25) | 63.4 | 100.0 |
| Sugar-sweetened beverages | Less-healthy plant foods | 2 / 4 / 2 | 3838 | 1.00 (1.00–1.50) | 0.0 | 0.0 |
| Sugar-sweetened beverages | Less-healthy plant foods | 3 / 3 / 3 | — | Not formed | — | — |
| Sugar-sweetened beverages | Less-healthy plant foods | 4 / 2 / 4 | — | Not formed | — | — |
| Sugar-sweetened beverages | Less-healthy plant foods | 5 / 1 / 5 | — | Not formed | — | — |
| Sweets and desserts | Less-healthy plant foods | 1 / 5 / 1 | 9615 | 0.25 (0.00–0.44) | 39.6 | 100.0 |
| Sweets and desserts | Less-healthy plant foods | 2 / 4 / 2 | 5527 | 0.83 (0.67–1.00) | 0.0 | 0.0 |
| Sweets and desserts | Less-healthy plant foods | 3 / 3 / 3 | 4361 | 1.50 (1.25–1.50) | 0.0 | 0.0 |
| Sweets and desserts | Less-healthy plant foods | 4 / 2 / 4 | 3957 | 2.40 (2.00–3.00) | 0.0 | 0.0 |
| Sweets and desserts | Less-healthy plant foods | 5 / 1 / 5 | — | Not formed | — | — |
| Red meat | Animal foods | 5 / 5 / 5 | 14933 | 0.00 (0.00–0.25) | 63.1 | 100.0 |
| Red meat | Animal foods | 4 / 4 / 4 | 5773 | 0.50 (0.50–0.60) | 0.0 | 0.0 |
| Red meat | Animal foods | 3 / 3 / 3 | 2754 | 1.00 (0.75–1.00) | 0.0 | 0.0 |
| Red meat | Animal foods | 2 / 2 / 2 | — | Not formed | — | — |
| Red meat | Animal foods | 1 / 1 / 1 | — | Not formed | — | — |
| Processed meat | Animal foods | 5 / 5 / 5 | 12842 | 0.00 (0.00–0.00) | 77.3 | 100.0 |
| Processed meat | Animal foods | 4 / 4 / 4 | 6871 | 0.67 (0.50–1.00) | 0.0 | 0.0 |
| Processed meat | Animal foods | 3 / 3 / 3 | 3747 | 1.50 (1.33–2.00) | 0.0 | 0.0 |
| Processed meat | Animal foods | 2 / 2 / 2 | — | Not formed | — | — |
| Processed meat | Animal foods | 1 / 1 / 1 | — | Not formed | — | — |
| Poultry | Animal foods | 5 / 5 / 5 | 10754 | 1.00 (1.00–1.00) | 0.0 | — |
| Poultry | Animal foods | 4 / 4 / 4 | 2074 | 2.00 (1.50–2.00) | 0.0 | — |
| Poultry | Animal foods | 3 / 3 / 3 | — | Not formed | — | — |
| Poultry | Animal foods | 2 / 2 / 2 | — | Not formed | — | — |
| Poultry | Animal foods | 1 / 1 / 1 | — | Not formed | — | — |
| Fish | Animal foods | 5 / 5 / 5 | 12558 | 0.00 (0.00–0.00) | 76.4 | 100.0 |
| Fish | Animal foods | 4 / 4 / 4 | 7392 | 0.50 (0.33–0.50) | 0.0 | 0.0 |
| Fish | Animal foods | 3 / 3 / 3 | 3510 | 1.00 (0.83–1.17) | 0.0 | 0.0 |
| Fish | Animal foods | 2 / 2 / 2 | — | Not formed | — | — |
| Fish | Animal foods | 1 / 1 / 1 | — | Not formed | — | — |
| Eggs | Animal foods | 5 / 5 / 5 | 19215 | 0.00 (0.00–0.25) | 64.6 | 100.0 |
| Eggs | Animal foods | 4 / 4 / 4 | 4245 | 1.00 (0.67–1.00) | 0.0 | 0.0 |
| Eggs | Animal foods | 3 / 3 / 3 | — | Not formed | — | — |
| Eggs | Animal foods | 2 / 2 / 2 | — | Not formed | — | — |
| Eggs | Animal foods | 1 / 1 / 1 | — | Not formed | — | — |
| Dairy | Animal foods | 5 / 5 / 5 | 7165 | 1.00 (0.50–1.00) | 0.0 | — |
| Dairy | Animal foods | 4 / 4 / 4 | 3957 | 1.50 (1.50–1.50) | 0.0 | — |
| Dairy | Animal foods | 3 / 3 / 3 | 4173 | 2.00 (2.00–2.00) | 0.0 | — |
| Dairy | Animal foods | 2 / 2 / 2 | 5413 | 3.00 (2.67–3.67) | 0.0 | — |
| Dairy | Animal foods | 1 / 1 / 1 | — | Not formed | — | — |

Notes: Q1–Q5 denote each food group's own original intake categories before domain-specific scoring; the uPDI points assigned to an observed category are shown in parentheses. Values are based on the final 23,460-participant Model 2 sample and are reported as n and median (interquartile range). The original scoring algorithm used empirical quintile cut points (R quantile type 7). Duplicate cut points caused by tied values were collapsed, and identical values were not split across categories; therefore, discrete or zero-inflated components could form fewer than five distinct categories. Points were assigned by category rank without rescaling (k points for the kth category of a positively scored component and 6 − k points for a reverse-scored component), so a component with fewer than five categories received only part of the 1–5 range. Had every component formed five categories, uPDI would have ranged from 15 to 75; given the categories shown, the attainable range was 35 to 69. The complete-component theoretical range was 15-75; the observed uPDI range in the analytic dataset was 14 to 49 because missing component scores were omitted from the sum, consistent with the prespecified scoring implementation. Dashes indicate categories that were not formed or quantities that were not applicable. For whole grains, legumes, refined grains, potatoes, poultry, and dairy, the component intake variable contained no zero values, and 9,217, 14,667, 2,530, 4,226, 10,632, and 2,752 participants, respectively, had no recorded intake; these participants are therefore not shown in the intake categories above, and the share of all non-consumers is not applicable for these components. **Supplementary Table S8. Correlation and collinearity diagnostics for uPDI and overall PDI**

| Analysis | n | Estimate | P value | Interpretation |
| --- | --- | --- | --- | --- |
| Pearson correlation: uPDI and overall PDI | 23460 | 0.555 | <0.001 | Linear correlation between standardized scores |
| Spearman correlation: uPDI and overall PDI | 23460 | 0.543 | <0.001 | Rank correlation between standardized scores |
| Variance inflation factor: uPDI | 23460 | 1.561 | — | Calculated against overall PDI and Model 2 covariates |
| Variance inflation factor: overall PDI | 23460 | 1.621 | — | Calculated against uPDI and Model 2 covariates |

Notes: uPDI, unhealthy plant-based diet index; PDI, overall plant-based diet index. Correlations used standardized scores in the final Model 2 sample. Each variance inflation factor was calculated by regressing the named score on the other score and all Model 2 covariates.

**Supplementary Table S9. Mutually adjusted uPDI and overall PDI imaging-wide association summary**

| Diet score | IDP category | No. IDPs | Nominal P <0.05 | Category FDR <0.05 | Global FDR <0.05 | Strongest associated IDP | Beta | P value |
| --- | --- | --- | --- | --- | --- | --- | --- | --- |
| Unhealthy plant-based diet index | other imaging phenotype | 20 | 5 | 0 | 0 | Mean rfMRI head motion, averaged across space and time points | 0.024 | 0.006 |
| Overall plant-based diet index | other imaging phenotype | 20 | 6 | 5 | 3 | Mean rfMRI head motion, averaged across space and time points | -0.077 | <0.001 |
| Unhealthy plant-based diet index | brain structural MRI volumes | 176 | 53 | 20 | 11 | Volume of grey matter (normalised for head size) | 0.029 | <0.001 |
| Overall plant-based diet index | brain structural MRI volumes | 176 | 53 | 35 | 31 | Volume of grey matter in Temporal Fusiform Cortex, anterior division (right) | 0.034 | <0.001 |
| Unhealthy plant-based diet index | brain functional MRI / T2star | 30 | 1 | 0 | 0 | 90th percentile of BOLD effect (in group-defined amygdala activation mask) for faces-shapes contrast | -0.019 | 0.048 |
| Overall plant-based diet index | brain functional MRI / T2star | 30 | 12 | 8 | 5 | Median T2star in hippocampus (left) | 0.032 | 0.001 |
| Unhealthy plant-based diet index | brain diffusion MRI / white-matter tracts | 675 | 88 | 1 | 4 | Mean MO in external capsule on FA skeleton (left) | 0.037 | <0.001 |
| Overall plant-based diet index | brain diffusion MRI / white-matter tracts | 675 | 182 | 42 | 58 | Mean ISOVF in body of corpus callosum on FA skeleton | -0.033 | <0.001 |

Notes: Models included standardized uPDI and standardized overall PDI simultaneously and adjusted for age, sex, ethnicity, household income, education, smoking, alcohol, physical activity, and total energy intake. Field 25000 (T1 volumetric scaling) was additionally included exactly as in the original imaging-wide analysis for all outcomes except field 25000 itself. Of 910 candidate IDPs, 901 had sufficient data for model estimation. Category and global FDR values used the Benjamini–Hochberg procedure. Complete IDP-specific estimates are provided in Supplementary Data S1.

**Supplementary Figure S1. Top hPDI-associated interpretable IDPs**

Supplementary Figure S1 shows top non-technical IDPs associated with hPDI.

**Supplementary Figure S2. hPDI and uPDI beta concordance**

Supplementary Figure S2 compares hPDI and uPDI beta estimates across brain IDPs.

**Supplementary Figure S3. hPDI and uPDI category heatmap**

Supplementary Figure S3 summarizes hPDI and uPDI associations by IDP category. Cell labels show the number of category-FDR significant IDPs and nominal associations.
